# Contribution of HIV Infection, AIDS, and Antiretroviral Therapy to Exocrine Pathogenesis in Salivary and Lacrimal Glands

**DOI:** 10.3390/ijms19092747

**Published:** 2018-09-13

**Authors:** Imran Nizamuddin, Peter Koulen, Carole P. McArthur

**Affiliations:** 1 School of Medicine, University of Missouri-Kansas City, Kansas City, MO 64108, USA; imrannizamuddin@mail.umkc.edu; 2 Vision Research Center, Department of Ophthalmology, School of Medicine, University of Missouri-Kansas City, Kansas City, MO 64108, USA; 3 Department of Biomedical Sciences, School of Medicine, University of Missouri Kansas City, Kansas City, MO 64108, USA; 4 Department of Oral and Craniofacial Sciences, School of Dentistry, University of Missouri-Kansas City, Kansas City, MO 64108, USA; McArthurC@umkc.edu

**Keywords:** acquired immune deficiency syndrome, ART, diffuse infiltrative lymphocytosis syndrome, DILS, dry eye disease, human immunodeficiency virus, Sjögren’s syndrome, xerophthalmia, xerostomia

## Abstract

The structure and function of exocrine glands are negatively affected by human immunodeficiency virus (HIV) infection and its co-morbidities, including innate and adaptive immune responses. At the same time, exocrine function may also be influenced by pharmacotherapies directed at the infectious agents. Here, we briefly review the role of the salivary glands and lacrimal glands in normal physiology and exocrine pathogenesis within the context of HIV infection and acquired immune deficiency syndrome (AIDS), including the contribution of antiretroviral therapies on both. Subsequently, we discuss the impact of HIV infection and the types of antiretroviral therapy on disease management and therapy development efforts.

## 1. Introduction

Exocrine glands produce and secrete onto epithelial surfaces by way of ducts. Two important exocrine glands are the salivary glands and lacrimal glands, which produce saliva and tears, respectively. These glands, as well as breast, exocrine pancreas, and prostatic tissues, share many commonalities in their overall function, histology, and mechanism of production and secretion of fluid [1]. Furthermore, they are often impacted simultaneously by disease processes and a myriad of medications.

### 1.1. Salivary Glands

Saliva is primarily produced and secreted by the three major salivary glands—the parotid, submandibular, and sublingual glands—with further contributions from minor salivary glands [1,2,3]. While these distinct glands may synthesize different proteins and components of saliva, they all work together in conjunction with the autonomic nervous system to formulate a heterogeneous fluid that has significant influence on human health. In this sense, salivary glands are unique among exocrine glands, as glands in different locations secrete into a common compartment and these individual secretions combine to form whole saliva [4].

Saliva serves an extensive number of purposes. It maintains a moist oral mucosa, making the oral cavity less susceptible to abrasions. Saliva contains mucins, which allow it to coat and lubricate the oral cavity during mastication, swallowing, and speaking. Additionally, saliva possesses various protective functions. Through its role in swallowing, it helps remove residual food and microbes within the oral cavity. Saliva also contains in excess of two thousand proteins and peptides with antibacterial, antiviral, and antifungal effects. These proteins assist in maintaining the oral microbiome to sustain oral health and prevent systemic infections. In addition, saliva forms the dental pellicle, the protein film that covers surface enamel, offering additional tooth protection against the development of caries. Furthermore, saliva is involved in the sensation of taste as it serves as a solvent in which food particles dissolve, begin digestion by salivary amylases, and interact with taste receptors. Evidently, saliva and its constituting proteins are critical for the proper functioning of multiple bodily tasks [5,6,7]. The numerous functions of saliva are outlined in Table 1 below.

The salivary glands are divided into lobules, which contain numerous acini. An individual acinus is lined by a layer of cuboidal epithelial cells surrounding the central lumen. The lumina merge to form intercalated ducts, which later become striated and interlobular ducts [8,9,10].

Depending on the gland, the histological composition of acini varies in the percentage of serous, muco-serous, and mucous acini, which are distinguishable following hematoxylin and eosin staining. Fluid secretion is triggered by an increase in intracellular calcium concentration, which activates chloride channels and leads to sodium and water movement [3,11,12]. Acinar cells initially produce isotonic fluid. However, this fluid is modified by ductal cells, which reabsorb most of the NaCl and secrete KHCO_3_. As a result, the fluid is rendered hypotonic as it flows to the mouth. The glands differ in terms of contributions to unstimulated saliva production compared with stimulated saliva production. Recent studies have suggested that these differences are related to the magnitude of Ca^2+^ signaling in response to stimulation and distinct activities of Cl^−^ transporters [4].

Salivary flow is under both direct and indirect control by the autonomic nervous system. Parasympathetic input is dependent on cholinergic signaling and varies based on the gland. For example, parasympathetic innervation to the sublingual and submandibular glands is supplied by the facial nerve (CN VII), while the parotid gland is supplied by the glossopharyngeal nerve (CN IX). In contrast, sympathetic innervation of all salivary glands is dependent on adrenergic signaling and is carried by postganglionic fibers originating from the superior cervical ganglion [13]. Parasympathetic stimulation typically evokes the release of high-flow, low-protein serous secretions, while sympathetic stimulation leads to the release of low-flow, high-protein mucinous secretions [8]. Salivary gland secretion is governed by a nerve-mediated reflex based on chemosensory, masticatory, and tactile stimulation; however, it is also modulated by nerve signals from other centers via neuro-hormonal influences [14].

As mentioned previously, the composition of saliva varies based on the gland of origin and is determined by the cellular Na^+^ gradient and acetylcholine signaling pathways. Typically, saliva is composed of water (~99.5%), electrolytes, antimicrobial factors, and various enzymes, among other important elements. Enzymes include amylase, lipase, lysozyme, and immunoglobulins [7,8].

### 1.2. Lacrimal Glands

The lacrimal gland is similar to salivary glands in its mechanism of contributing to ocular surface health. Lacrimal secretions allow for the creation of the human tear film, made of the lipid, aqueous, and mucous layers. The tear film lubricates the surface of the eye, functions as a barrier against foreign body and microbial invasion, and supplies the avascular cornea with nutrients and oxygen [15]. The gland produces many proteins and aqueous fluid to add volume to the tear film. Furthermore, the lacrimal gland also secretes several bactericidal and fungicidal agents, akin to the salivary glands.

On the other hand, the lacrimal gland is made of several lobules separated by loose connective tissue. Each lobule consists of many acini, lined with columnar serous cells that produce a watery secretion. The central lumina of many units converge to form intralobular ducts, which unite to drain into 8–12 excretory ducts [16,17]. The lacrimal gland is supplied by parasympathetic innervation via the facial nerve (CN VII). Preganglionic fibers originate in the lacrimal nucleus in the pons (a portion of the superior salivatory nucleus), synapse in the pterygopalatine ganglion, and then travel with the lacrimal nerve, the smallest branch of CN V_1_. In contrast, sympathetic innervation of the lacrimal gland is similar to the salivary glands in that postganglionic fibers originate from the superior cervical ganglion. These fibers travel along the internal carotid artery plexus to the pterygopalatine ganglion, where they join with parasympathetic fibers to the lacrimal gland [18].

## 2. Mechanism of Fluid Secretion

Research over the last several decades has established that Ca^2+^ is the primary intracellular factor that regulates fluid secretion by exocrine glands [11,19,20]. Ca^2+^ regulates ion fluxes that allow for the creation of an osmotic gradient to drive fluid secretion and electrolyte accumulation in the lumina [21,22]. Recent research has suggested that the depletion of calcium stores in the endoplasmic reticulum (ER) triggers store-operated Ca^2+^ entry (SOCE), whereby stromal interaction molecule 1 (STIM1), a calcium sensor in the ER, activates plasma membrane ion channels made of Orai subunits, leading to an increase in intracellular calcium [23].

A number of plasma membrane receptors are involved in Ca^2+^ mediated signaling cascades. Salivary glands have muscarinic, alpha-adrenergic, and purinergic receptors, while lacrimal glands have muscarinic, melanotropin, and adenylyl cyclase-activated VIP receptors to which neurotransmitters may bind [24,25]. Receptor activation triggers a G_αq/11_-protein mediated cascade, activating phospholipase C, leading to the hydrolysis of phosphatidylinositol 4,5-bisphosphonate (PIP_2_), and ultimately increasing inositol triphosphate (IP_3_) concentrations. IP_3_ binds to receptors (mainly IP_3_R2 and IP_3_R3) on the ER, resulting in rapid release of Ca^2+^ stores into the cytosol. This process is highly regulated with feedback and feed-forward regulation by cytosolic Ca^2+^ levels. Ultimately, calcium release leads to the activation of various ion channels and triggers fluid secretion. Sustained secretion, however, requires calcium influx. Studies have suggested that in salivary glands, this Ca^2+^ entry is mediated by store-operated calcium entry (SOCE). The molecular components of the SOCE channel have yet to be completely elucidated, but current models suggest communication between ER Ca^2+^ stores and the plasma membrane, which plays a role in calcium entry from the extracellular space. Studies have shown that transient receptor potential channel 1 (TRPC1) is a major contributor to SOCE in salivary gland acinar cells and that it has interactions with STIM1 and Orai1 [11,13,20,22,23,26,27].

Traditionally, most studies of exocrine gland molecular signaling pathways involving Ca^2+^ have been based on salivary and pancreatic cell models. Nevertheless, newer studies further demonstrate that, even in lacrimal acini, Ca^2+^ entry is mediated by SOCE through the phospholipase C-IP_3_ pathway. Similar to salivary gland signaling pathways, studies also highlight the role of Orai1 in SOCE in the lacrimal gland [22,28].

## 3. In Vitro Salivary Models

Numerous models are currently in use to study both the histological and physiological characteristics of salivary glands (Table 2). Nevertheless, at the moment, no cell line is able to fully emulate the structure and function of native salivary acinar cells. Tumor-derived cell lines include A, 253, Nagoya-78, 563, human submandibular gland cell line (HSG), human parotid epithelial cell line (HSY), rat submandibular gland acinar epithelial cell line (SMIE), and rat submandibular duct epithelial cell line (RSMT-A5) [29,30]. 

HSG is a neoplastic cell line that was established from an irradiated human submandibular gland. Morphologically, the cells are cuboidal and form glandular-like structures that resemble intercalated duct cells. HSG cells have specializations, such as junctional complexes connecting neighboring cells, and intercellular digitations formed by papillary infoldings. Furthermore, HSG cells have cytoplasmic organelles, including Golgi complexes and rough endoplasmic reticulum, suggesting exocytotic ability [31,32]. HSG cells are commonly used as in vitro models for salivary function for several reasons [33]. They develop an acinar phenotype and express amylase when cultured on Matrigel. Cell proliferation can be controlled by regulators of apoptosis, such as cimetidine. Moreover, HSG cells can be used to study muscarinic and purinergic input as functional receptors are present and coupled to calcium signaling. A drawback is their inability to form tight junctions on plastic, and, as a result, they cannot achieve the polarity needed for fluid secretion [29].

HSY is a human parotid gland adenocarcinoma cell line derived from athymic mice [34,35]. Morphologically, these cells resemble intercalated duct cells, but also have characteristics of myoepithelial cells. A number of features make HSY cells an important in vitro salivary model. HSY cells exhibit tight junctions and other intercellular junctions required to maintain a polarized monolayer organization, which is essential for fluid secretion. They are able to express amylase, necessary for replicating the function of salivary gland tissues [36]. Moreover, HSY cells respond to autonomic agonists to increase intracellular levels of Ca^2+^ and cAMP. This rise is necessary for the secretion of saliva in vivo. Furthermore, the growth and differentiation of HSY cells can be easily modulated by transfection [29,37].

SMIE and RSMT are cell lines derived from rat submandibular glands [29,38]. Structurally, SMIE cells appear relatively undifferentiated. Due to a low expression of claudin-3, SMIE cells possess a leaky epithelium with low measurements of transepithelial electrical resistance and relative permeability to molecules, such as dextran and mannitol [39]. As a result, SMIE is considered useful for studying osmotic transepithelial fluid movement and polarity. RSMT-A5 cells also originate from the submandibular gland and display a ductal epithelium phenotype [40,41]. They cannot achieve polarity and thus do not secrete fluid. Furthermore, RSMT-A5 cells do not express amylase. They could potentially be used to study cell signaling due to the high density of α1-adrenergic receptors; however, protein secretion studies are not plausible as these cells are difficult to transfect [29,42].

Several immortalized cell lines are also used to study salivary glands and include rat submandibular gland epithelial cell lines (SMG-C) and rat parotid gland cell lines (PAR-C). SMG-C6 and SMG-C10 are highly-differentiated acinar cell lines that were established following transfection of rat submandibular acinar cells by an *SV40* genome [43]. These cell lines have a secretory function and can polarize by forming tight junctions and desmosomes. SMG-C6 cells are excellent models for intracellular calcium signaling as Ca^2+^ release can be stimulated by muscarinic and purinergic receptor pathways via SOCE [44]. Both SMG-C6 and SMG-C10 also respond to β-adrenergic agonists. Their properties and response to glucocorticoid and mineralocorticoid modulation make these cell lines ideal for the investigation of Na^+^ channels and the expression of ENaC [45]. In contrast, Par-C are acinar cell lines that were established following transfection of rat parotid glands by an *SV40* genome. This cell line can form secretory granules, intracellular connections, and microvilli. No amylase expression is noted [46]. In both Par-C5 and Par-C10 cell lines, [Ca^2+^]_i_ is regulated by cholinergic, muscarinic, and α1-adrenergic agonists [47]. Par-C10 is a good model to characterize secretion, as many studies have characterized its transepithelial anion secretion and proteins on its basolateral surface [48].

## 4. Salivary and Lacrimal Pathology

Dry mouth syndrome (xerostomia) and dry eye disease (keratoconjunctivitis sicca) are common outpatient complaints and often occur together, referred to as the “sicca complex”. Some studies suggest that up to 25% of patients who visit eye clinics report symptoms of dry eyes [49]. One study reported that among 2481 elderly patients, 27% reported either dry eye or dry mouth symptoms, and 4.4% reported both [50]. Nevertheless, due to the diverse etiology of such symptoms, clinicians often encounter difficulty in attributing a single underlying cause. Although dry eyes and dry mouth are often attributed to Sjögren’s syndrome, sicca symptoms can also be secondary to other autoimmune diseases, diabetes, environmental factors, infection, graft-versus-host disease, or drug side effects. 

Xerostomia refers to the subjective complaint of dryness of the oral cavity. Nevertheless, xerostomia does not always correlate to objective salivary gland hypofunction, which can be measured by salivary flow rates. Many investigators define the criteria for salivary hypofunction using cutoffs of 0.1 mL/min and 0.7 mL/min for unstimulated and chewing-stimulated salivary flow rates, respectively. Submandibular glands are the main contributors of unstimulated salivary secretions, while parotid glands contribute to over half of the salivary flow of stimulated secretions [51]. Low salivary flow rates can lead to a number of conditions, including dysphagia, dysgeusia, dental caries, and periodontal disease [52,53,54,55].

Dry eye disease is characterized by deficient tear production or excessive tear evaporation, resulting in ocular irritation. Several medication conditions, including blepharitis and autoimmune destruction of lacrimal glands, can result in damage to lacrimal acini or excretory ducts, leading to dry eye disease [18]. Dry eye syndromes also have tear film instability due to a number of factors, resulting in dysfunction of the lacrimal functional unit [56]. Treatment is essential to prevent sight-threatening complications, such as corneal ulcers or infections. 

Human immunodeficiency virus (HIV) infection has been associated with a number of glandular manifestations leading to sicca symptoms. In this review, we will highlight HIV-associated salivary and lacrimal gland disease, as well as sequelae of antiretroviral therapy (ART) that may lead to glandular signs and symptoms. In addition, diffuse infiltrative lymphocytosis syndrome (DILS) will be explored as a consequence of HIV infection. 

## 5. Sequelae of HIV (Human Immunodeficiency Virus) and Antiretroviral Drugs on Salivary Glands

Oral manifestations related to HIV reportedly occur in 30–80% of HIV patients and are often the presenting signs of infection [57,58,59,60]. These not only include neoplasms, such as Kaposi’s sarcoma, and opportunistic infections, such as oral candidiasis and oral hairy leukoplakia, but also HIV-associated salivary gland disease (HIV-SGD). First noted by Schiodt et al. in 1989, HIV-SGD typically refers to lymphocytic infiltration of the salivary glands in consequence of HIV infection [61]. Over time, long-standing HIV infection of the salivary glands may result in the development of benign lymphoepithelial lesions, which contain epimyoepithelial islands and an extensive lymphoid infiltrate [62,63]. Consequently, this lymphoid proliferation leads to salivary gland enlargement and xerostomia [60]. HIV-associated benign lymphoepithelial cysts can be confirmed by HIV-1 p24 antigen immunohistochemical staining [64]. Furthermore, increased CD8^+^ expression in the interfollicular areas may indicate possible HIV-related lymphoepithelial lesions compared to other cystic parotid lesions [65]. Of note, patients with HIV-SGD are at an increased risk for non-Hodgkin lymphomas (44× increased risk compared to the general population), specifically mucosa-associated lymphoid tissue lymphoma [66,67].

The introduction of ART has led to an overall decrease in oral complications secondary to HIV infection. Nevertheless, the prevalence of HIV-SGD is less clear due to a lack of recent studies. Some studies suggest that the prevalence of HIV-SGD has increased [68,69]. For example, one study carried out in HIV/acquired immune deficiency syndrome (AIDS) patients in Cameroon demonstrated that 57 of the 59 patients studied had lymphocytic infiltrates in biopsies of minor salivary glands. None of the patients had bilateral parotid swelling or were receiving ART, suggesting that if parotid swelling is applied as a criterion to diagnose HIV-SGD, many patients could be over-looked [70]. On the other hand, other studies indicate that ART reduces the incidence of HIV-SGD [71]. One study suggested that the prevalence of DILS in subjects tested in Houston, Texas declined from 4% prior to therapy to 0.8% after the introduction of therapy [72]. Another study from Greece found that the prevalence of glandular manifestations prior to therapy was 7.8% and decreased after the introduction of ART [73]. These conflicting results may be due to differences in medication regimens, ethnic differences, or therapy duration. Patients in resource-limited environments, for example, often receive nucleoside reverse transcriptase inhibitors (NRTIs) and non-nucleoside reverse transcriptase inhibitors (NNRTIs). On the other hand, patients in the USA and Europe often receive a regimen that includes NRTIs plus a protease inhibitor (PI) or integrase inhibitor (INI). There are no reports to date of the prevalence of SGD in HIV patients receiving INIs, which >80% of patients in the USA currently receive [74]. Furthermore, confusion arises due to the lack of a consensus definition and diagnostic criteria for HIV-SGD.

The pathogenesis of HIV-SGD is currently unknown. In an attempt to explain the rising prevalence of HIV-SGD in the ART era, many investigators hypothesized its etiology as an immune reconstitution inflammatory syndrome. If this hypothesis were correct, ART use leading to reconstitution of antigen-specific immune responses could lead to an “unmasking” of an underlying opportunistic infection, resulting in salivary gland disease. Viral etiologies are plausible and are currently being investigated. One study found that HIV-SGD was more prevalent in children within their sample, which could indicate that primary viral infection in children may be linked to HIV-SGD [75]. Some have proposed that an opportunistic infection by BK polyomavirus is involved, since mice infected with polyomavirus have developed enlarged parotid glands and even malignancy [76]. Jeffers et al. detected high salivary levels of BK virus DNA in patients with HIV-SGD. Furthermore, they demonstrated the ability for BK virus to infect and replicate within salivary gland cells in vitro [59,77]. While the pathogenesis of HIV-SGD remains unclear, further investigations of other potential viral etiologies are eagerly awaited.

With the introduction of ART, there has been a decreased frequency in oral manifestations of HIV by an estimated 10–50%, especially for oral candidiasis [78]. Yet, studies have demonstrated the ability of ART, like many other drugs, to contribute to xerostomia and hyposalivation. Even so, it remains difficult to distinguish the adverse effects of ART and HIV-related oral disease manifestations. 

Certain NRTIs, such as didanosine, have been associated with subjective xerostomia [79,80,81]. Moreover, PIs specifically have been linked to decreased salivary flow rates (both stimulated and unstimulated) compared with non-PI based ART in HIV patients [82,83]. While the mechanism remains unclear, it is hypothesized that PIs may cause an alteration in the structure and composition of saliva, leading to a decreased salivary flow. Olive et al. further suggested that PIs can lead to abnormal fat accumulation and parotid lipomatosis, thus causing enlarged salivary glands and reduced salivary flow [84]. Still, establishing a link between genes regulating lipid metabolism and HIV-1 protease gene remains difficult. Furthermore, a Mexican study has suggested that ART, regardless of CD4^+^ counts, served as a probable risk factor for decreased salivary flow and hyposalivation based on the number of years of therapy. This study did not find PI use to have a significant impact on salivary dysfunction in patients on ART [85]. Studies on newer combination agents for ART are lacking.

Meanwhile, certain ART drugs are also known to cause subjective xerostomia (dry mouth) as a side effect, yet do not objectively affect salivary gland function. Studies have suggested that patient complaint of xerostomia does not necessarily indicate diminished salivary gland function [86,87]. Future researchers should clearly report the individual medications comprising ART in future studies.

## 6. Sequelae of HIV and Antiretroviral Drugs on Lacrimal Glands

HIV infection has numerous ophthalmic manifestations, ranging from underlying microvasculopathy to opportunistic infections to autoimmune reactions [88,89]. These complications are described as common, even affecting up to 50–75% of HIV-infected individuals [90]. They may involve virtually every component of the eye, including the orbit and adnexal structures, the anterior segment, and the posterior segment. While posterior segment lesions may have greater ocular morbidity, anterior segment lesions, such as keratoconjunctivitis sicca, are common and can have a substantial impact on quality of life [90]. The HIV-1 virus and HIV RNA has been reported to persist in the tears of affected patients even when ART reduces the viral load to undetectable levels [91]. 

The etiology of dry eye disease in HIV patients is usually thought to be due to HIV-mediated lymphocytic infiltration of the lacrimal gland, although many opportunistic infections and underlying conditions may contribute to worsening symptoms. This leads to the destruction of lacrimal acini and the ductal system, as well as direct conjunctival damage [92]. The resulting keratoconjunctivitis contributes to a chronic inflammatory state, further promoting cytokine secretion, destruction and dysfunction of the lacrimal gland, and loss of tear production [18]. The exact mechanism of decreased tear production in keratoconjunctivitis remains unknown, but it is known that the secretion of cytokines impedes the neural transmission that normally results in tear production. Investigation is currently underway regarding specific cytokine networks, and the cytokine profile may be different depending on the underlying cause [93]. The loss of neural support promotes glandular atrophy, and cellular breakdown proteins go to the cell surface and further activate T-cells. Increased expression of intercellular adhesion molecule 1 (ICAM-1) and lymphocyte function-associated antigen 1 (LFA-1) in keratoconjunctivitis, causing homing and antigen presentation of T-cells, is known to be important [94,95,96]. Recently, LFA-1/ICAM-1 interaction has been investigated as a potential therapeutic target [97]. Interferon-gamma is also suspected to have an important role in immunopathogenesis [98].

The inciting factor for lymphocytic infiltration of the lacrimal gland remains unclear. Some researchers have studied viral etiologies, but findings have yet to demonstrate a significant correlation between dry eyes and the detection of viral DNA (Herpesviruses) in conjunctival and tear secretions of HIV-positive individuals [99].

While tear film abnormalities may be due to either direct infiltration of the lacrimal gland by HIV or an alteration of the tear film layers as a byproduct of ART, differentiating the two etiologies is difficult. Few studies have been conducted with the purpose of studying potential lacrimal side effects of ART. Even though ART is a documented cause of xerostomia, its contribution toward xerophthalmia in HIV patients remains unclear. 

Moreover, the prevalence of dry eye disease among HIV patients is unclear, with estimates ranging from 20% to 85% of HIV-positive patients [15,100]. Keratoconjunctivitis is typically assessed using various tests, including TFBUT (tear film break-up time), the Schirmer wetting test, vital staining with Rose Bengal and sodium fluorescein, and the mean tear osmolarity test. One study of long-term survivors of perinatally-acquired HIV infection found abnormal Schirmer test results in only 9% of patients [101]. On the other hand, another study found that the TFBUT was abnormal in 70% of patients and tear IgE was increased in 36% of patients [102]. The impact of ART on the prevalence of dry eye disease is also controversial, with studies reaching differing conclusions [103,104,105]. One recent study found that patients on long-term ART (>36 months), compared with short-term ART (<12 months), were more likely to have clinically significant keratoconjunctivitis sicca based on TFBUT [106]. Another study found that the incidence of tear film abnormalities has not changed in the post-ART era compared to the pre-ART era [107,108]. Differences in conclusions may be due to varying medication regimens or differing characteristics in populations sampled.

Overall, while HIV infection and antiretroviral drugs are both known to contribute to dry eye disease, further investigation must be done to establish prevalence and investigate the underlying pathophysiology. ART-related structural changes of the lacrimal gland have not been studied and can be an avenue of further research.

## 7. Diffuse Infiltrative Lymphocytosis Syndrome: Definition and Differential Diagnoses

As stated above, HIV infection commonly leads to both salivary and lacrimal glandular dysfunction, leading to dry mouth and dry eye diseases. Since the common etiology of these diseases is lymphocytic infiltration, a syndrome referred to as diffuse infiltrative lymphocytosis syndrome (DILS) has been proposed to describe the systemic dysfunction of multiple glands as a result of HIV infection. DILS is defined as an HIV-associated disease that causes destruction of glands, namely the salivary and lacrimal exocrine glands, due to glandular CD8^+^ T cell infiltration in the setting of reduced CD4^+^ counts [71,72,73,109,110,111,112]. Some studies have suggested that DILS affects 3–7.8% of HIV-infected patients [72,111]. Worldwide, its prevalence is reportedly highest in Africa, while in the United States, DILS is most prevalent in African Americans [109,112].

DILS was initially identified in 1985 as lymph node hyperplasia and parotid gland enlargement in HIV-positive patients [113]. Later, in 1989, this complex was coined “diffuse infiltrative lymphocytosis syndrome” [114]. Researchers then found that certain human leukocyte antigen (HLA) class I haplotypes, such as HLA-B45 and HLA-B49, conferred an increased risk of developing DILS [115]. Early criteria proposed by Itescu for the diagnosis of DILS required salivary gland enlargement or xerostomia for >6 months and lymphocytic infiltration of the affected gland on biopsy in HIV-confirmed patients [116]. Table 3 below highlights the suggested diagnostic criteria.

With the introduction of ART, the epidemiology, clinical presentation, and extra-glandular manifestations have become more complex, but evidence suggests that DILS is a host humoral response to HIV antigens [71]. Some have speculated that the decreasing prevalence of DILS may be due to ART [112], leading to decreased circulating CD8^+^ lymphocytes [111,117]. A recent study by Chen et al. in Taiwan noted that ART decreased the risk of DILS overall. In this study, lopinavir was associated with a decreased risk of DILS, while zalcitabine was associated with an increased risk [118]. Therefore, it is important to know if an HIV-positive patient is receiving ART prior to making this diagnosis. 

Clinical manifestations of DILS consist of bilateral parotiditis (with parotid enlargement and lymphadenopathy) and sicca symptoms, such as xerostomia and xerophthalmia. DILS may be further accompanied by a range of extra-glandular organ involvement [109,112]. Lymphocytic interstitial pneumonia can present with a dry cough and progressive exertional dyspnea [119,120,121]. Peripheral axonal neuropathy may occur due to endoneural and perineural CD8^+^ infiltration, and aseptic meningitis may also be present. The neuropathy, acute or subacute in onset, typically begins with painful paresthesias in the feet followed by sensorimotor loss predominantly involving the lower limbs. Reflexes are lost in the affected nerve distribution. While cranial nerve involvement is uncommon, facial nerve palsy may be present [122,123,124]. 

Furthermore, DILS can lead to lymphocytic interstitial nephropathy, lymphocytic hepatitis, cystitis, and gastrointestinal infiltration. In all of these cases, the organs are infiltrated by CD8^+^ lymphocytes [71,111]. While the underlying pathophysiology of DILS is yet to be elucidated, studies hypothesize that infected lymphocytes secrete cytokines that stimulate endothelial and ductal cells to produce surface markers, including ICAM-1. These markers stimulate the migration of activated CD8^+^ lymphocytes to the sites of antigenic invasion [33,116].

DILS may be mistaken for a number of other conditions that also cause sicca symptoms, the most common being Sjögren’s syndrome (SS). DILS and SS are compared in Table 4 below. A patient with SS often presents initially to an oral health professional with various complaints, including xerostomia, periodontal disease, gingivitis, or keratoconjunctivitis sicca. Upon further questioning, the female patient may also admit to dryness of the skin and vagina. Systemic involvement of the lungs, liver, vasculature, and kidneys may also be present. Histologically, SS involves the focal infiltration of T cells, B cells, and plasma cells around the glandular ducts of exocrine glands. The histological picture of SS and its effect on glands is superficially indistinguishable from DILS, but must be differentiated based on laboratory testing for HIV/AIDS and autoantibodies [125,126,127,128]. Testing is typically done for several autoantibodies, such as anti-nuclear antibody (ANA), SS-A (Ro), SS-B (La), and/or rheumatoid factor (RF), which may be positive in patients with SS. Furthermore, compared to DILS, in SS, HLA haplotypes tend to be distinctive and the CD4^+^/CD8^+^ ratio is usually normal [116]. Recently, the role of osteopontin in contributing to SS pathogenesis has been investigated [129]. On the other hand, plasma osteopontin levels were previously suggested to be elevated in HIV patients despite ART [130]. Further investigation of these interlinking pathways may provide valuable discoveries regarding the pathogenesis of both SS and DILS.

IgG4-related disease may also involve the salivary and lacrimal glands (IgG4-related sialadenitis), leading to a similar clinical presentation. Similar to DILS, IgG4-related disease results in lymphadenopathy and can lead to extra-glandular involvements, including tubulo-interstitial nephropathy, interstitial pneumonitis, sclerosing pancreatitis, sclerosing cholangitis, and retroperitoneal fibrosis. The condition normally presents with cervical lymphadenopathy and enlargement of the parotid or sublingual glands. It must be differentiated from DILS based on elevated levels of IgG4 and IgE, as well as the presence of CD4^+^ and CD25^+^ T_reg_ cells at the site of infiltration [131,132]. All in all, DILS is a difficult diagnosis usually made upon the basis of patient symptoms in conjunction with HIV-testing.

Other viral infections may elicit sicca symptoms as well. For example, chronic infection by the hepatitis C virus (HCV) is known to induce a number of extrahepatic and rheumatological manifestations [133]. The prevalence of sicca symptoms has been estimated to affect between 10% to 30% of HCV-infected patients [134,135,136,137,138]. On the other hand, less than 5% of patients with SS are HCV-positive [133]. Some studies report that up to 50% of all HCV patients have chronic focal sialoadenitis [139]. As a result, sicca symptoms are common in this population, though usually less severe when compared to SS [137,140,141]. It remains unknown whether the virus causes a disease that mimics SS, or if HCV leads to the development of SS. Of note, although sicca symptoms are common in patients infected with HCV, a typical SS characterized by the presence of SS-A and SS-B antibodies is rare [133]. Epstein-Barr virus (EBV) is often also associated with various autoimmune disorders, including SS, leading to autoimmune exocrinopathy [142,143]. Moreover, cytomegalovirus (CMV) infection of ductal cells of the salivary and lacrimal glands can lead to altered cell surface antigenic expression, resulting in tissue destruction. Destruction of salivary acinar cells and salivary ducts leads to xerostomia and sicca symptoms [54,144]. Studies have suggested that the detection of EBV, CMV, or HCV in saliva in HIV patients did not decrease saliva or tear production [145]. As a result, the salivary gland disease and eye disease associated with HIV is likely unrelated to Sjögren’s syndrome and autoimmune causes, although HIV has an autoimmune component evidenced by immunodeficiency.

## 8. Current Approaches to Exocrine Gland Dysfunction as a Consequence of HIV Infection and Antiretroviral Therapy

The current therapeutic approach towards management of DILS centers on the use of ART. The prevalence of DILS has decreased since the advent of ART, which suggests ART plays a role in disrupting chronic antigen stimulation that leads to elevated CD8^+^ lymphocyte levels [71,111,147]. Many studies further demonstrate that the institution of ART has improved CD8^+^ lymphocytosis and led to reversal of visceral infiltration [71,111,148,149]. One study suggested that ART was effective in resolving lymphoepithelial parotid cysts [150]. Likely, patients will have already started treatment for HIV infection; thus, ART should be maintained and optimized.

In the acute setting, steroids may be useful for patients with significant symptoms. Oral prednisone, with doses ranging from 15 mg to 60 mg daily, can be given for six to eight weeks, after which the dosage is tapered. Nevertheless, further studies must be conducted to objectively assess the impact of steroid use [124,147,151]. Some reports suggest that low-dose radiotherapy may be effective on a short-term basis in reducing parotid gland enlargement in DILS patients [152,153].

Other than managing the underlying infection, symptomatic control of keratoconjunctivitis sicca is important in mitigating the effects of exocrine gland dysfunction as a consequence of infection and ART. Table 5 below highlights current treatment strategies.

### 8.1. Dry Mouth Disease

For dry mouth, treatment strategies are aimed at compensating for the loss of the normal salivary functions mentioned above. The main principles of treatment involve stimulating existing salivary flow, replacing salivary secretions, and paying close attention to dental care and infections [154,155,156].

Patient self-care is an important aspect of preventing dryness. Studies have suggested maintaining good hydration (i.e., regular sips of water), avoiding oral irritants and acidic drinks, avoiding medications that may worsen oral dryness (i.e., anticholinergics), and avoiding low-humidity environments [157,158,159]. Patients may also replace oral secretions using water, ice, or artificial saliva [155]. In addition, regular dental care is important as dry mouth predisposes the development of dental caries, especially root and incisal caries [160]. Guidelines suggest meticulous self-care, regular dental checkups (at least once every six months), plaque control, and avoiding sugary snacks [155,157]. Special toothpastes are available for patients with dry mouth, which lack detergents that can further irritate the dry mouth [161].

In addition to the measures listed above, patients with dry mouth also benefit from topical stimulation of salivary flow. A number of products may be used, ranging from gums to candies to dried fruit slices [155,157,162]. Nevertheless, research has not shown a clear advantage of one product over another [157]. Limited research has suggested utilization of a topically applied oral insert that may stimulate salivary flow, but further studies must be conducted to assess its effectiveness [163,164].

If a patient does not respond adequately to the basic measures, artificial saliva and muscarinic agonists can be considered. Regarding artificial saliva, products differ in viscosity and other characteristics [165]. Therefore, response may vary from patient to patient. Several observational studies show the benefit of artificial saliva in relieving dry mouth symptoms. Nevertheless, they have not been shown in randomized trials to improve salivary flow [157,166,167]. One study suggested both symptomatic control and improvement in salivary flow with the use of an oral hydroxycellulose spray [157,168].

Other than artificial saliva, muscarinic agonists, including pilocarpine and cevimeline, are recommended in patients who continue to have dry mouth symptoms even with topical stimulants or saliva replacement. Several randomized trials have shown the benefits of both pilocarpine and cevimeline in increasing salivary flow and improving dry mouth symptoms. These drugs appear to be equally efficacious compared to placebo, although no studies have compared their effects head-to-head [169,170,171,172,173,174,175,176,177]. The choice between the two is usually due to individual factors, including cost, convenience, and other side effects, etc. Notably, since pilocarpine is a non-selective muscarinic agonist, it may also help with dry eye symptoms, although studies have not noted changes in tear production [175]. Due to side effects, some patients may prefer to discontinue use of muscarinic agonists if a sufficient response is not present. Oral candidiasis should be excluded if patients have an inadequate symptomatic response.

Several systemic anti-inflammatory or immunosuppressive agents, such as hydroxychloroquine, rituximab, and infliximab, have been explored in patients with Sjögren’s syndrome to treat dry mouth symptoms [178,179,180]. However, these drugs are not typically used for dry mouth disease caused by DILS.

### 8.2. Dry Eye Disease

Current treatment guidelines for dry eye disease suggest a number of environmental and pharmacological approaches that can be initiated. Initial management highlights the importance of lifestyle changes, such as avoiding dry environments; as well as avoiding long exposure to computers, TV, and reading. Dietary consumption of omega-3 fatty acids may also work to promote anti-inflammatory effects [181]. Furthermore, elimination of offending systemic medications is important, as hundreds of medications may be implicated in both dry eye and dry mouth disease. While dry eyes as a consequence of ART may be unavoidable, the use of other medications that may exacerbate the problem should be avoided.

First line treatment for dry eye disease usually involves the use of hypromellose-containing artificial tear substitutes. These drops function by moisturizing the ocular surface, thus alleviating complaints of dry eyes. Other lubricating eye drops may also treat dryness by diluting irritating agents and fortifying layers of the tear film [182]. Studies have reported that frequent use of these substitutes may reduce signs of corneal damage [183]. Nevertheless, the overall impact of artificial tears is limited, as they lack the beneficial anti-inflammatory growth factors and cytokines of human tears [181]. Most lubricating eye drops also contain preservatives, which have the potential to irritate the eye. While clinical studies regarding the impact of preservatives have been mixed, guidelines suggest that preservative-free eye drops are preferred for those requiring drops over four times per day [181,183].

Newer therapies for the management of dry eyes involve anti-inflammatory agents (including topical corticosteroids, topical cyclosporine, and topical or systemic omega-3 fatty acids), tetracyclines, punctual plugs, and secretagogues. As dry eye is now known to be a chronic inflammatory disease mediated by T-cell activation and cytokine production as mentioned above, anti-inflammatory agents may represent a major advance in dry eye therapy [184]. Corticosteroids initiate an immunosuppressive effect by promoting the expression of anti-inflammatory genes in leukocytes and preventing the expression of pro-inflammatory genes. However, long term use has the potential to contribute to ocular hypertension [182]. Topical cyclosporine has been shown to have benefits in subjective and objective measures of dry eye with limited adverse effects, especially in patients with SS [185,186]. Lifitegrast is a newer topical agent that inhibits LFA-1 and prevents binding of ICAM-1, thus inhibiting inflammatory pathways. Head-to-head comparisons with cyclosporine are lacking, although some have suggested that lifitegrast may have more rapid clinical improvement and few adverse effects [187]. Topical nonsteroidal anti-inflammatory drugs (NSAIDs) are not typically used—while they may reduce ocular surface inflammation, they have not been shown to be consistently effective. They are also associated with several corneal complications, i.e., irritation, keratitis, corneal melting, and corneal perforation. Tetracycline derivatives are typically used for dry eye disease caused by Meibomian gland dysfunction. Secretagogues, such as diquafosol tetrasodium, and bromhexine, improve mucin secretion overall to reduce surface inflammation and tear film instability. Overall, these agents have varied mechanisms of action, but possess anti-inflammatory effects and improve the symptoms of ocular surface disease [182]. They may be helpful in treating dry eye disease associated with DILS, although no studies have specifically assessed the symptomatic control of dry eye disease in DILS patients.

Autologous serum eye drops have also been used to manage severe cases of dry eye disease. The rationale behind its use is that human serum contains many important constituents of the tear film, including growth factors, fibronectin, and lysozyme. Thus, autologous serum provides key factors to optimize the ocular surface [188]. Autologous serum eye drops are gaining popularity based on recent studies that have reported promising results [189,190]. Nevertheless, a Cochrane review found no evidence of a long-term effect compared to artificial tears and recommended large randomized-controlled trials to assess its benefit [191]. Overall, the treatment for dry eye disease, regardless of cause, currently involves lifestyle modification and the use of lubricating eye drops, along with consideration of newer therapies if necessary.

## 9. Conclusions

This review encompassed a wide variety of topics, ranging from normal salivary/lacrimal function to pathology linked to HIV/AIDS and medication side effects to treatment options. The salivary and lacrimal glands are both exocrine glands that produce saliva and tears, respectively, which are vital for proper organ function. They both share a common mechanism for fluid secretion, and as a result, these glands are often linked in systemic pathologies. Thus, glandular dysfunction can lead to dry eye disease or dry mouth disease, which may greatly affect overall quality of life. While there are many causes for this glandular dysfunction, this review focuses on DILS, a complication of HIV, which leads to CD8^+^ infiltration of various glands. Studies have not recommended additional treatment for DILS other than prompt initiation of ART and symptomatic control. Unfortunately, several antiretroviral drugs have also been associated with glandular dysfunction, which obviously presents a dilemma.

While DILS was first reported in 1985, the literature still does not describe significant breakthroughs regarding this disorder. Many studies are now dated and randomized control trials are still lacking. Further investigation is warranted into a number of topics, including the mechanism of DILS and the signaling cascade involved in cellular localization; antiretroviral-related structural changes of exocrine glands; the effects of modern antiretroviral drugs; and treatment options for DILS (including steroid use or novel applications of symptomatic control). Furthermore, most studies addressing symptomatic control of dry eye and dry mouth symptoms are specific to SS; thus, investigation is needed regarding treatment of dry eye and dry mouth disease in patients with DILS and other causes of glandular dysfunction.

## Figures and Tables

**Table 1:** Overview of the functions of saliva. Adapted from Humphrey and Williamson, 2001 [3].

Function	Principal Salivary Components Involved
lubrication	mucin glycoproteins
digestion	α-amylase
microbial control	IgA, IgG, IgM, mucins, lactoferrin, lysozyme, peroxidase
pH maintenance	bicarbonate phosphates, urea
protection of teeth (pellicle formation)	macromolecular proteins, stratherins, histatins, cystatins, proline-rich proteins
gustation	H_2_O (as solvent), protein, gustin, zinc

**Table 2:** Current cell models used to study the characteristics of salivary glands. Adapted from Nelson et al. [29].

Name	Source	Ability to Achieve Polarity	Amylase Expression	Ability to Secrete Fluid	References
HSG	irradiated human submandibular gland	(+)	(+)	(+)	Shirasuna et al. (1981) [31], Shirasuna et al. (1986) [32]
HSY	human parotid gland adenocarcinoma derived from athymic mice	(+)	(+)	(+)	Yanagawa et al. (1986) [35]
SMIE	rat submandibular gland, immortalized with retrovirus vector	(+)	(−)	(+)	He et al. (1990) [38], He et al. (1998) [39]
RSMT-A5	rat submandibular gland, immortalized by methylcholanthrene	(−)	(−)	(−)	Brown (1973) [40], He et al. (1989) [41]
SMG-C	rat submandibular gland, immortalized by *SV40* genome	(+)	Unknown	Unknown	Quissell et al. (1997) [43]
Par-C	rat parotid gland, immortalized by *SV40* genome	(+)	(−)	(+)	Quissell et al. (1998) [46]

**Table 3:** Diagnostic criteria for DILS (diffuse infiltrative lymphocytosis syndrome), suggested by Itescu et al. (requires all criteria) [116].

Diagnostic Criteria for DILS
1. HIV infection (positive serology) 2. Bilateral salivary gland enlargement or xerostomia 3. Persistence of signs/symptoms for 6 months or more 4. Histologic confirmation of salivary or lacrimal gland lymphocytic infiltration without granulomatosis or neoplastic involvement

**Table 4:** Comparison of key characteristics of DILS (diffuse infiltrative lymphocytosis syndrome) and SS (Sjögren’s syndrome). Both are characterized by focal lymphocytic sialoadenitis [146].

Category	Diffuse Infiltrative Lymphocytosis Syndrome	Sjögren’s Syndrome
association	HIV/AIDS	Autoimmune diseases
histology	glandular CD8^+^ T cell infiltration	primarily CD4^+^ T cell infiltration (staining not usually performed)
clinical manifestations	parotiditis xerostomia xerotoconjunctivitis sicca hypergammaglobulinemia possible extraglandular organ involvement	usually no parotiditis xerostomia keratoconjuncitivits sicca hypergammaglobulinemia possible extraglandular organ involvement
laboratory tests supporting diagnosis	HIV seropositivity autoantibodies rarely present	autoantibodies including ANA, SS-A (Ro), SS-B (La), RF
HLA association	DR5 (DR11), DR6 (DR13)	B8, DR2, DR3, DR4, DQ2, A1
treatment	ART, corticosteroids, symptomatic treatment	symptomatic treatment

**Table 5:** Current approaches to exocrine gland dysfunction as a consequence of DILS.

Category	Treatment Options
general	antiretroviral therapy (ART) consider corticosteroid use
dry mouth disease	lifestyle modifications artificial saliva substitutes muscarinic agonists
dry eye disease	lifestyle modifications artificial tear substitutes anti-inflammatory agents (topical corticosteroids, cyclosporine, lifitegrast) autologous serum eye drops

## Abbreviations

AIDS	acquired immune deficiency syndrome
ANA	anti-nuclear antibody
ART	antiretroviral therapy
DILS	diffuse infiltrative lymphocytosis syndrome
EBV	Epstein-Barr virus
HCV	hepatitis C virus
HIV	human immunodeficiency virus
HIV-SGD	HIV-associated salivary gland disease
SOCE	store-operated Ca^2+^ entry
SS	Sjögren’s syndrome
TFBUT	tear film break-up time
